# Antioxidant Activity and Effects of Protamex-Assisted Protein Hydrolysates from *Trichiurus lepturus* By-Products in a Dexamethasone-Induced C2C12 Muscle Atrophy Model

**DOI:** 10.3390/antiox15101320

**Published:** 2026-10-13

**Authors:** Cheol-Yeong Yoo, Rajasinghe Peli Gedara Sewwandi Kaushalya Amarasiri, Sang-Woon Lee, Jeong Min Lee, Mi-Jin Yim, Hyun-Soo Kim

**Affiliations:** 1 Department of Seafood Science and Technology, Gyeongsang National University, Tongyeong 53064, Republic of Korea; cjfdud7104@gnu.ac.kr; 2 Department of Marine Life Sciences, Jeju National University, Jeju 63243, Republic of Korea; sewwandi1995@office.jejunu.ac.kr (R.P.G.S.K.A.); swlee@office.jejunu.ac.kr (S.-W.L.); 3 National Marine Biodiversity Institute of Korea, Seochun 33662, Republic of Korea; lshjm@mabik.re.kr

**Keywords:** protein hydrolysate, oxidative stress, myotube, dexamethasone-induced atrophy, fish by-product valorization

## Abstract

Hairtail (*Trichiurus lepturus*) is a popular fish species in South Korea that generates substantial quantities of processing byproducts. This study investigated the potential functional properties of hairtail byproducts to enhance their utilization. Proximate composition analysis showed that crude protein was the most abundant component, accounting for approximately half of the total composition. Therefore, protein hydrolysates were prepared from hairtail byproducts using Protamex-assisted enzymatic hydrolysis. The yield of Protamex-assisted enzymatic hydrolysate (HBP) produced based on the raw material drying weight was 42.8%. Amino acid analysis revealed high levels of Aspartic acid and glutamic acid, which can provide instrumental help in muscle synthesis and growth, and free amino acids showed high L-leucine content. HBP also exhibited considerable antioxidant activity. In a dexamethasone-induced C2C12 cell atrophy model, HBP increased cell viability in a concentration-dependent manner, reaching 120.58% at 300 µg/mL. Cell proliferation also increased in a concentration-dependent manner, reaching 125.54% at the same concentration. Immunofluorescence analysis of myosin heavy chain (MyHC) and DAPI staining further indicated concentration-dependent enhancement of muscle cell differentiation. These findings suggest that hairtail byproducts may provide a sustainable source of functional protein hydrolysates with potential protective effects against muscle atrophy in a dexamethasone-induced C2C12 model.

## 17. Introduction

Fish processing generates byproducts such as heads, vertebrae, skin, viscera, and tails, which account for approximately 30–75% of the raw material during fileting and cutting [1]. A substantial proportion of these byproducts are landfilled, discarded at sea, incinerated, or used as low-value feed ingredients, resulting in environmental and economic concerns. With increasing emphasis on the sustainable use of marine resources and the circular economy, considerable research has focused on converting marine byproducts into high-value functional materials [2]. Fish byproducts are promising sources of functional food ingredients because they contain proteins, including collagen, gelatin, and bioactive peptides, as well as other bioactive compounds such as omega-3 fatty acids and chitosan [3,4,5].

The high protein content of fish byproducts makes them suitable raw materials for the production of bioactive compounds [2]. Enzymatic hydrolysis is particularly advantageous because it can be performed under mild reaction conditions and may result in less amino acid loss than chemical hydrolysis. Enzyme specificity can also influence the molecular characteristics and biological activities of the resulting peptides [6,7,8]. However, the bioactivity of protein hydrolysates is also shaped by hydrolysis-derived structural factors, as the degree of hydrolysis, resulting peptide molecular weight, and amino acid sequence collectively determine their structure–activity relationships [9,10]. Fish protein hydrolysates exhibit various biological activities, including antioxidant, anti-inflammatory, antihypertensive, anti-obesity, anticancer, and immunomodulatory effects, supporting their potential application as functional food ingredients.

Hairtail (*Trichiurus lepturus*) is widely distributed in tropical and temperate waters worldwide and belongs to the order Scombriformes and family Trichiuridae [11]. In Korea, hairtail ranked third among coastal fishery species in annual catch from 2020 to 2024, and 37 of the 41 coastal fisheries identified hairtail as a major target species [12]. The annual hairtail catch reached a low of 32,000 tons in 2011 but subsequently recovered to 60,000 tons in 2023 [7]. Protein hydrolysates derived from hairtail have been reported to exhibit antioxidant, antihypertensive, and anti-fatigue activities, increasing interest in their potential use as functional food ingredients [13,14,15]. However, compared with other fish species, the functional properties of protein hydrolysates derived from hairtail head and skeletal byproducts remain insufficiently characterized, particularly with respect to their potential effects on muscle atrophy [5].

The antioxidant activity of protein hydrolysates may be closely associated with the maintenance of muscle health and prevention of muscle atrophy [16]. Sarcopenia and muscle atrophy can occur in response to aging, malnutrition, muscle disuse, septicemia, cachexia, metabolic acidosis, and insulin deficiency and are associated with alterations in glucocorticoid signaling and oxidative stress [7,17]. It should be noted that muscle atrophy is a broad term encompassing muscle mass loss from various causes, whereas sarcopenia specifically refers to age-related decline in muscle mass and strength; thus, not all muscle atrophy constitutes sarcopenia [18]. Excessive production of reactive oxygen species (ROS) can contribute to muscle atrophy by impairing muscle protein synthesis and activating proteolytic pathways, including the ubiquitin-proteasome system. Dexamethasone, a synthetic glucocorticoid, is commonly used experimentally to induce muscle atrophy through mechanisms that include oxidative stress and enhanced protein degradation. Accordingly, dexamethasone-treated C2C12 myotube cells are widely used as an in vitro model for investigating muscle atrophy and sarcopenia [19]. The dexamethasone-induced C2C12 model used in the present study represents a glucocorticoid-induced muscle atrophy model rather than a model of sarcopenia.

The antioxidant properties of protein hydrolysates are partly influenced by their amino acid composition. Aromatic amino acids, such as tryptophan, tyrosine, and phenylalanine, can contribute to antioxidant activity through electron- or hydrogen-donating properties associated with their indole or aromatic side chains. Such amino acids are therefore frequently found in peptides with antioxidant activity. By reducing oxidative stress, antioxidant peptides may attenuate ROS-mediated pathways involved in muscle protein degradation. In addition to antioxidant properties, the biological activities of protein hydrolysates may be influenced by their overall amino acid composition. Other studies have reported that glutamine supplementation can promote muscle protein synthesis (MPS), reduce muscle protein breakdown (MPB), speed up wound healing, and alleviate muscle injury in animal experiments [20]. Thus, characterization of the amino acid composition of protein hydrolysates may provide insight into their potential muscle-related functional effects. Furthermore, naturally derived protein hydrolysates have been investigated for their potential to reduce muscle atrophy.

Therefore, this study investigated the potential utilization of hairtail processing byproducts as a source of functional protein hydrolysates. We hypothesized that HBP would attenuate dexamethasone-induced morphological and biological impairment in C2C12 myotubes, an effect potentially related to its antioxidant activity, without inferring the specific molecular mechanism underlying this relationship. To this end, the proximate composition of hairtail byproducts was characterized, and protein hydrolysates were prepared from the head and vertebrae using Protamex-assisted enzymatic hydrolysis. The amino acid composition and antioxidant activity of the resulting hydrolysates were evaluated, and their potential protective effects against dexamethasone-induced muscle atrophy—assessed primarily by cell viability, myotube morphology, and indices of myogenic differentiation—were examined as the primary endpoint of this study.

## 2. Materials and Methods

### 2.1. Reagents

Required chemicals and mixtures were procured from Sigma-Aldrich (St. Louis, MO, USA) unless otherwise indicated. Goat anti-mouse IgG H&L (Alexa Fluor™ Plus 555; A32727, Thermo Fisher Scientific Inc., Waltham, MA, USA) for immunofluorescence was obtained from Invitrogen (Cambridge, UK). Commercially available condensed maca (*Lepidium meyenii*), which is used in food supplements, was purchased from NOW Foods (Lot No. 3063322, Bloomingdale, IL, USA) and used as the positive control [21].

### 2.2. Protein Hydrolysis

Hairtail byproducts, including the head, vertebrae, and tail, were obtained in Jeju-si, Jeju-do, Republic of Korea, after removal of the edible portions and viscera. The hairtail byproducts (head, vertebrae, and tail) were freeze-dried and ground using a blender. Distilled water (50 mL, *w/v* per gram of sample powder), followed by addition of 1% (*w/v*) Protamex (Sigma-Aldrich, St. Louis, MO, USA). Enzymatic hydrolysis was performed at 100 rpm, pH 6, and 37 °C for 24 h. The reaction mixture was heated at 90 °C for 15 min to inactivate the enzyme. The resulting protein hydrolysate was filtered through filter paper (No. 2 Qualitative Filter Paper, ADVANTEC, Tokyo, Japan) and used for subsequent experiments.

### 2.3. Proximate Composition Analysis

The proximate composition of hairtail byproducts was analyzed according to AOAC methods (AOAC International, 2005) [22]. Moisture content was determined using the atmospheric-pressure oven-drying method, crude protein using the Kjeldahl method, crude fat content using Soxhlet extraction with ether, and ash content by incineration in a furnace at 550 °C [23,24].

### 2.4. Constituent Amino Acid Composition Analyses

For the constituent amino acids, the sample was prepared so that the actual crude protein content was 3 mg/mL, then 3 mL of 12N HCl was added, and the mixture was placed in an airtight container and hydrolyzed at 115 °C for 24 h. After that, the sample was prepared by titrating with 4.2 N NaOH until the pH reached 2.5, then volumetrically added to a standard flask to a final volume of 50 mL, and filtered through a 0.2 μm microfilter. Constituent Amino acids were detected at a wavelength of 570 nm or 440 nm, with peak area quantification performed using the instrument’s OpenLAB CDS EZChrom Edition software (Agilent Technologies, Santa Clara, CA, USA). Using the Bio-chrom 30 Plus analyzer (Harvard Bioscience, Holliston, MA, USA) via a ninhydrin reaction. Quantification was performed using a single-point external calibration against a physiological amino acid standard mixture analyzed under identical chromatographic conditions to the samples: 10.000 nmol/40 µL for most amino acids and 5.000 nmol/40 µL for cystine. The amino acid content was expressed as g per 100 g crude protein [25].

### 2.5. Free Amino Acid Composition Analyses

Free amino acids were prepared by centrifuging 5× *g* of the sample diluted in 20% TCA at 8000 rpm for 20 min, then centrifuging the residue again with 10% TCA, and purifying the filtered supernatant. Subsequently, the lipids were removed using ether with a fractionation column. After concentrating it, 25 mL was adjusted with a lithium buffer and purified; then the sample was prepared by filtering through a 0.2 μm microfilter. Amino acids were detected at a wavelength of 570 nm or 440 nm, with peak area quantification performed using the instrument’s OpenLAB CDS EZChrom Edition software. Using the Bio-chrom 30 Plus analyzer (Harvard Bioscience, Holliston, MA, USA) via a ninhydrin reaction. Quantification was performed using a single-point external calibration against a physiological amino acid standard mixture analyzed under identical chromatographic conditions to the samples: 10.000 nmol/40 µL for most amino acids; 5.000 nmol/40 µL for L-cystine and cystathionine. The amino acid content was expressed in g per 100 g crude protein [25].

### 2.6. ABTS Radical-Scavenging Activity

ABTS radical-scavenging activity was measured using a method modified from [26]. Briefly, 180 µL ABTS reagent was mixed with 20 µL sample in a 96-well plate. After incubation for 5 min, absorbance was measured at 734 nm using a microplate reader.

For verification, the ABTS radical-scavenging formula and blank-correction method used were:

ABTS radical-scavenging activity (%) = [(Control – (Sample – Blank))/Control] × 100

Control: is the absorbance of ABTS solution without sample;

Sample: is the absorbance of ABTS solution with the test sample;

Blank: is the absorbance of the sample alone (without ABTS) to correct for intrinsic sample color/turbidity.

### 2.7. DPPH Radical-Scavenging Activity

DPPH radical-scavenging activity was measured using a modified method reported previously [26]. Briefly, 100 μL DPPH reagent was mixed with 100 μL sample and incubated in the dark for 30 min. Absorbance was subsequently measured at 517 nm using a microplate reader.

For verification, the DPPH radical-scavenging formula and blank-correction method used were:

DPPH radical-scavenging activity (%) = [(Control – (Sample – Blank))/Control] × 100

Control: is the absorbance of DPPH solution without sample;

Sample: is the absorbance of DPPH solution with the test sample;

Blank: is the absorbance of the sample alone (without DPPH) to correct for intrinsic sample color/turbidity.

### 2.8. C2C12 Cell Culture and Dexamethasone Exposure During Myogenic Differentiation

C2C12 myoblasts were purchased from American Type Culture Collection (ATCC, Manassas, VA, USA). Cells were cultured in Dulbecco’s modified Eagle’s medium (DMEM; WELGENE Inc., Gyeongsan-si, Gyeongsangbuk-do, Republic of Korea) containing 10% fetal bovine serum (FBS; WELGENE Inc.) and 1% penicillin and streptomycin (Invitrogen, Waltham, MA, USA) at 37 °C in a 5% CO_2_ humidified incubator (60915672, Sanyo Electric Co., Ltd., Osaka, Japan). Once cultures reached 70–80% confluence, cells were seeded at 1 × 10^5^ cells/well and cultured for 48 h. Cell differentiation was then induced by replacing the culture medium with DMEM containing 2% horse serum (HS; (HS; Gibco, Thermo Fisher Scientific, Waltham, MA, USA) Gibco, Invitrogen Inc.) and antibiotics. HBP was added at the onset of differentiation, and 10 μM dexamethasone was added 1 h later. The differentiation medium containing HBP and dexamethasone was renewed every 48 h for three consecutive treatment cycles, for a total concurrent differentiation and dexamethasone-exposure period of 8 days. Because HBP and dexamethasone were introduced at the onset of differentiation and maintained throughout this period, the design does not assess reversal of established atrophy in fully differentiated, mature myotubes. Rather, it evaluates whether HBP attenuates dexamethasone-associated impairment of myogenic differentiation and myotube formation during concurrent exposure.

### 2.9. Evaluation of Cytotoxicity

The cytotoxicity of HBP was measured using the MTT (3-(4,5-dimethylthiazol-2-yl)-2,5-diphenyltetrazolium bromide) assay [27]. C2C12 myoblasts were seeded into 96-well plates at 1 × 10^5^ cells/well and cultured for 24 h. Cells were then treated with HBP (10, 30, 100, and 300 µg/mL), followed 1 h later by 10 μM dexamethasone, and incubated for a further 24 h (48 h total). Following this treatment, 20 μL of 5 mg/mL MTT reagent in phosphate-buffered saline (PBS) was added to each well and incubated at 37 °C for 1 h in the presence of 5% CO_2_. The solution was then removed from all wells, and 100 μL DMSO was added to solubilize the formazan formed. Absorbance was measured at 540 nm using a microplate reader (Synergy™ HT, Agilent Technologies, Santa Clara, CA, USA).

### 2.10. Evaluation of Cell Proliferation

The effect of HBP on the proliferation of C2C12 myoblasts was evaluated using bromodeoxyuridine (BrdU) labeling and a detection kit (11647229001, Roche^®^ Life Science, Penzberg, Upper Bavaria, Germany) according to the vendor’s instructions. Cells were treated as described in Section 2.9. BrdU labeling and detection were then performed according to the manufacturer’s instructions.

### 2.11. Immunofluorescence Analysis

Differentiated myotubes were washed with cold PBS, fixed, and permeabilized with chilled methanol, followed by washing with phosphate-buffered saline containing Tween-20 (PBST) three times. Fixed and permeabilized cells were blocked with 1% bovine serum albumin (BSA) containing 22.52 mg/mL glycine for 2 h and incubated with a MyHC-specific primary antibody (Cat No # SC376157 from Santa Cruz Biotechnology, Dallas, TX, USA; 1:250 in 1% BSA) for 2 h at room temperature (25 °C). Cells were then incubated with goat anti-mouse IgG H&L (Alexa Fluor™ Plus 555; A32727, Thermo Fisher Scientific Inc., Waltham, MA, USA)-conjugated secondary antibody. Cell nuclei were stained with 300 nM 4′,6-diamidino-2-phenylindole (DAPI; Sigma-Aldrich). Fluorescence images were acquired using a confocal microscope (Carl Zeiss, Oberkochen, Germany). Three independently acquired replicate images were analyzed for each treatment group (*n* = 3), with one image corresponding to one replicate. Within each replicate, the MyHC, DAPI, and merged views represented the same microscopic field. Myotube diameter, nuclei within the MyHC-positive mask, fusion index, and myotube coverage were quantified using Myotube Analyzer software (Version 1.0.1) [28]. Each replicate image was analyzed separately, and the resulting image-level value for each endpoint was used as one replicate in the subsequent statistical analysis. Individual cells, nuclei, or myotubes detected within an image were not treated as independent statistical units.

### 2.12. Statistical Analysis

All data were analyzed using GraphPad Prism 8.0.2 (GraphPad Software Inc., San Diego, CA, USA) and are presented as the mean ± standard deviation (SD). For comparisons between two groups, Student’s t-test was used. For the image-derived endpoints, three independently acquired replicate images were analyzed for each treatment group (*n* = 3), and one image-level value for each endpoint was entered into the statistical analysis. The three replicate values per group were compared using one-way analysis of variance (ANOVA) followed by Dunnett’s multiple-comparisons test, with the dexamethasone-treated group used as the reference group. Individual cells, nuclei, and myotubes detected within an image were not treated as separate statistical replicates. A *p*-value < 0.05 was considered statistically significant. On rechecking the retained analysis records, a formal normality test and a separate homogeneity-of-variance test could not be verified; therefore, the previous unsupported statement that the data were normally distributed was removed.

## 3. Results

### 3.1. Proximate Composition and Hydrolysate Yield

The proximate composition of the freeze-dried hairtail byproducts used to prepare hydrolysates is presented in Table 1. Crude protein was the predominant component, accounting for 53.58 ± 1.80%, followed by crude fat 19.96 ± 0.77%.

According to previous studies, protein accounted for 47.2% of the proximate composition in bigeye tuna, 46.4% in salmon vertebra, 46.6% in mackerel vertebra, and 51.1% in herring vertebra [29,30].

**Figure 1:**
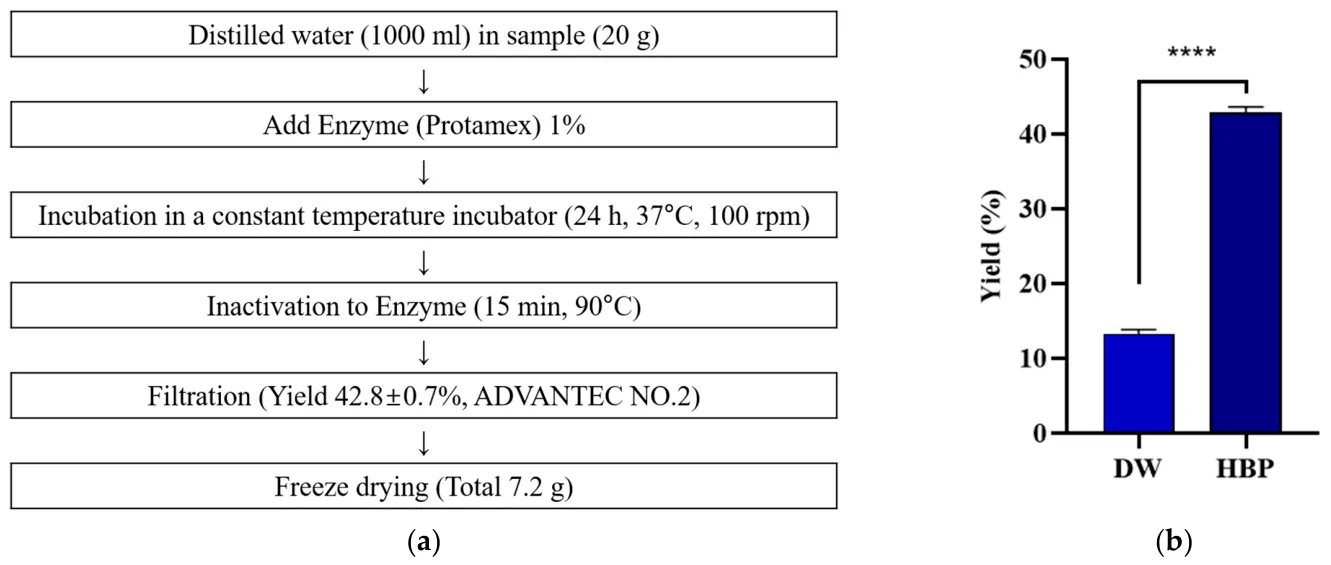
(**a**) By-product hydrolysis process of enzyme-assisted hairtail (*T. lepturus*), (**b**) Yield comparison between water extract and Protamex-assisted enzyme extract (HBP). Data expressed as the mean ± SD, using a t-test. Significant differences were identified at **** *p* < 0.0001 as compared to the DW control. All experiments were performed in triplicate (*n* = 3).

The extraction yield was 13.2 ± 0.58% for the water extract and 42.8 ± 0.79% for the Protamex-assisted hydrolysate (HBP) (Figure 1). The yield of HBP was significantly higher than that of the water extract. This demonstrates that when using Protamex-assisted enzymatic hydrolysis, the yield is more than three times higher than that obtained by conventional water extraction, showing that functional protein materials can be efficiently recovered from hairtail by-products. Such high yields can increase the amount of product recoverable per unit of raw material, serving as a positive factor in terms of productivity. The crude protein content of the dried HBP was 82.4 ± 0.27%.

### 3.2. Constituent and Free Amino Acid Composition

Among the constituent amino acids in HBP, Aspartic acid was the most abundant (15.82%), followed by glutamic acid (15.66%) (Table 2). Next were arginine, glycine, and proline. Although aspartic acid is not considered a classical antioxidant per se, it can indirectly contribute to antioxidant defense through the tricarboxylic acid cycle [31]. HBP contained glutamic acid at a relatively high ratio of 15.66% of the constituent amino acids, which serves as a direct precursor for glutamine synthesis in skeletal muscle through glutamine synthase [32]. Proline is a major constituent amino acid of collagen and contributes to maintenance and tissue regeneration in extracellular connective tissue. This amino acid is associated with the recovery and function of muscle. The extracellular matrix (ECM) controls muscle development by forming a three-dimensional support structure in muscle fibers and is linked to tendons, which are essential for muscle contraction and force transmission. Therefore, a high proline content may promote tissue recovery [33,34,35]. It has been reported that if proline supply through intake is insufficient, the synthesis of connective tissue proteins in the muscle may not increase. Therefore, abundant proline in HBP can help promote protein synthesis in muscle connective tissue through sufficient proline supply [36].

Leucine is a branched-chain essential amino acid involved in muscle function control amino acid promotes muscle protein synthesis by activating the mTOR signaling pathway [37,38]. The proportion of essential amino acids among constituent amino acids of HBP was 30.47%.

In free amino acid analysis, L-leucine accounted for 11.97%, whereas taurine accounted for 6.36% (Table 3). Essential amino acids accounted for 36.98% of the free amino acid composition. By selectively cleaving specific peptide bonds, enzymatic hydrolysis enables the degradation of a protein structure in a regulated manner while preserving the structure of individual amino acids. As a result, the nutritional value of hydrolyzed products may be preserved or even improved [39]. Furthermore, enzymatic hydrolysis produces a mixture of oligopeptides, low-molecular-weight peptides, and free amino acids, all of which show improved solubility, digestibility, and bioavailability compared with intact proteins [40]. The free amino acid form of leucine can be quickly absorbed without digestion and suppli These functional characteristics are closely related to muscle health. When gastrointestinal absorption rate increases, postprandial blood amino acid concentrations rise more rapidly and distinctly, which is very important for maximizing stimulation of muscle protein synthesis. Leucine is absorbed into the bloodstream and taken up by skeletal muscle, where it activates the mTORC1 pathway to induce muscle protein synthesis [35]. Free amino acids are absorbed more rapidly than intact proteins [34], resulting in a faster rise in blood essential amino acid levels, particularly leucine, following consumption; however, this faster absorption kinetics does not necessarily translate into a higher rate of muscle protein synthesis compared with intact protein sources. Our results suggest that HBP warrants further investigation as a candidate functional protein ingredient for supporting muscle health.

### 3.3. Antioxidant Activity

**Figure 2:**
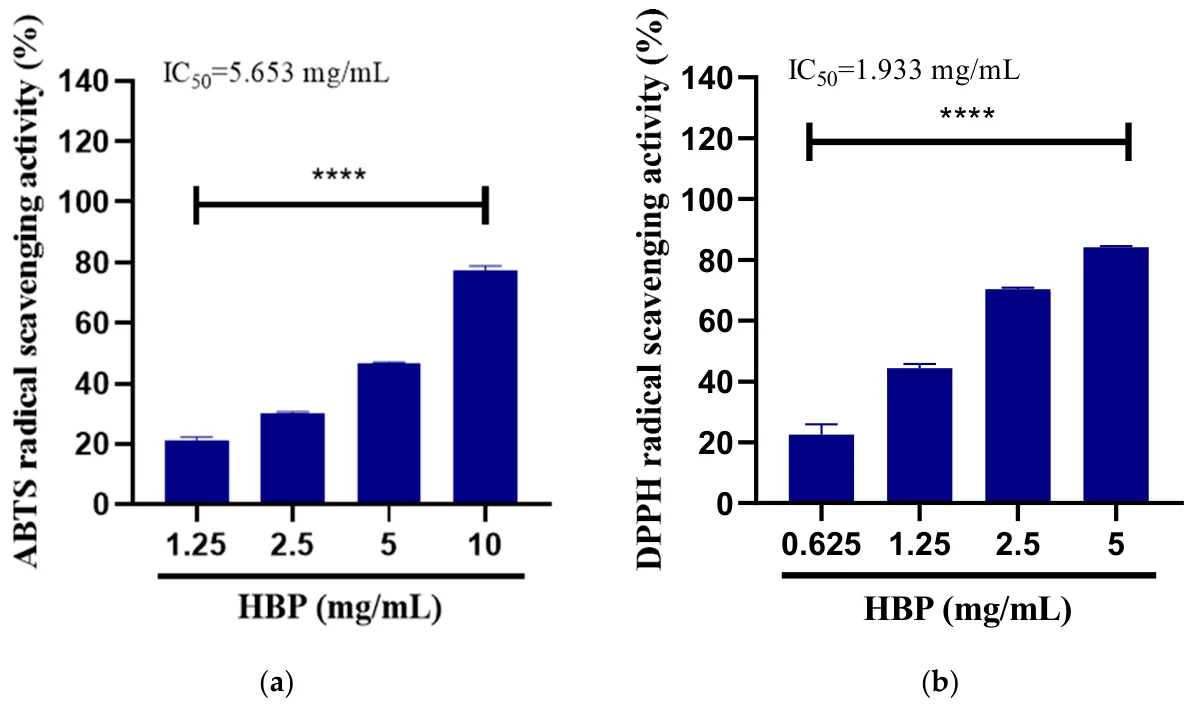
Measurement of antioxidant radical scavenging activity: (**a**) ABTS radical scavenging activity according to HBP concentration; (**b**) DPPH radical scavenging activity according to HBP concentration. Data expressed as the mean ± SD. Significant differences were identified at **** *p* < 0.0001 as compared to the Control.

HBP exhibited concentration-dependent ABTS radical scavenging activity, with a half-maximal inhibitory concentration of 5.653 mg/mL. HBP also exhibited DPPH radical scavenging activity, with a half-maximal inhibitory concentration of 1.933 mg/mL (Figure 2). According to a previous study, ABTS and DPPH results of hydrolysates of cod, herring, and mackerel have been reported, and although their IC_50_ values for DPPH varied, the herring protein hydrolysate was the lowest at 0.73 ± 0.11, while the highest value was reported for the cod-derived water-soluble protein hydrolysate fraction at 22.82 ± 1.10. ABTS showed that the herring protein hydrolysate fraction had the lowest value at 2.76 ± 0.05, while the cod-derived water-soluble protein hydrolysate fraction had the highest value at 125.25 ± 7.22. However, in that study, most of the hydrolyzed substances that showed efficacy were reported to be around 3 mg/mL and 10 mg/mL [41]. These figures are comparable to or lower than the values reported for protein hydrolysates derived from other species and by-products, suggesting that HBP possesses relatively superior antioxidant activity.

### 3.4. Cell Viability and Proliferation

In dexamethasone-treated C2C12 cells, no cytotoxicity was observed following treatment with HBP concentrations of 30, 100, or 300 µg/mL. Instead, cell viability increased in a concentration-dependent manner, reaching a maximum of 120.58% at 300 µg/mL. Also, no significant difference was observed in the positive control 100 µg/mL Maca group, which was used as the benign control group.

**Figure 3:**
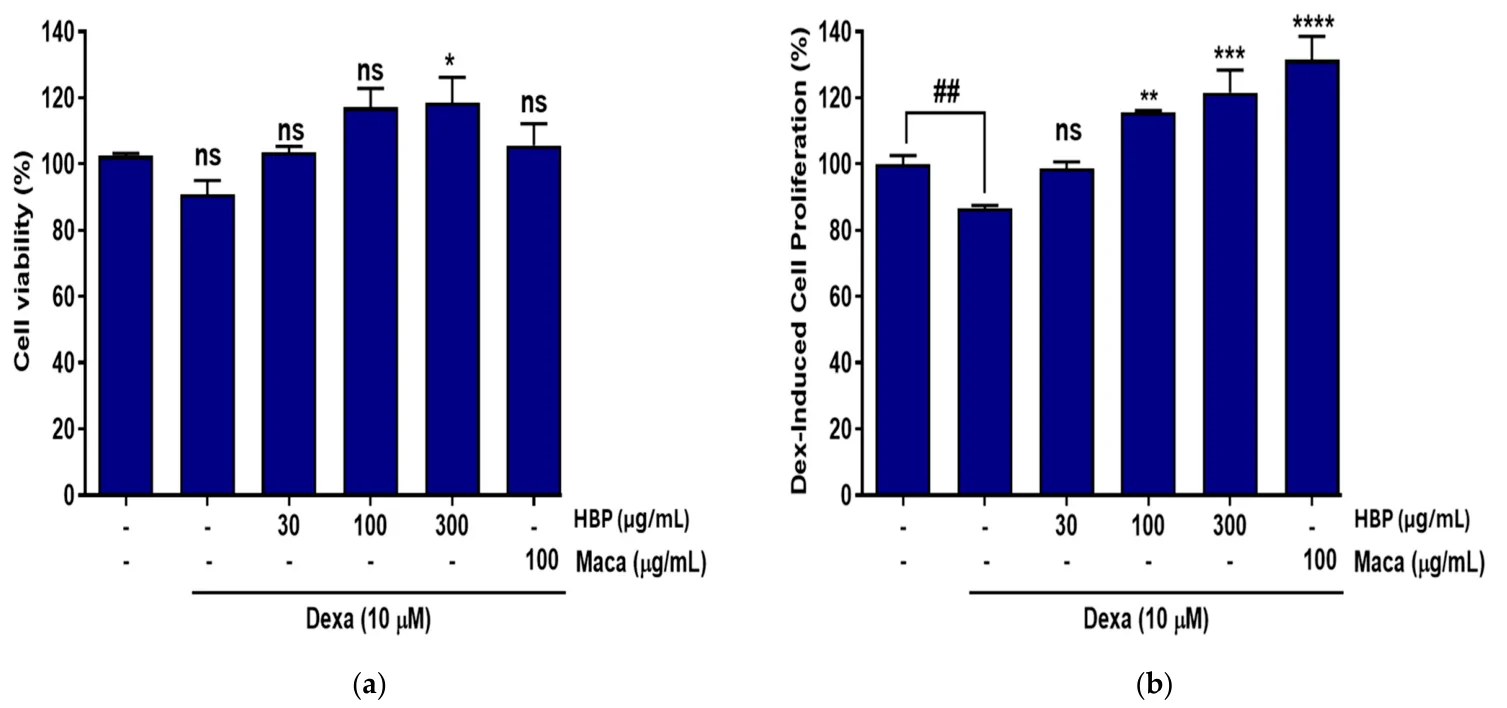
Experimental results of HBP in a dexamethasone-induced C2C12 myotube atrophy model of C2C12 cells induced by dexamethasone (Dexa): (**a**) Cell viability (**b**) Cell proliferation rate. Data expressed as the mean ± SD. Significant differences were identified at * *p* < 0.05, ** *p* < 0.01, *** *p* < 0.001 and **** *p* < 0.0001 as compared to the HBP control; ## *p* < 0.01 as compared to the DEXA-induced control; ns means are not significant.

Based on these findings, the same HBP concentrations were used in subsequent experiments. In the case of cell proliferation rate, Treatment with HBP at 30, 100, and 300 µg/mL also resulted in a concentration-dependent increase in cell proliferation (Figure 3). Cell proliferation increased with concentration, reaching 125.54% at the same concentration. In the cell proliferation assay, the Maca group was used as the positive control (100 µg/mL) and produced the greatest response, reaching 131.4%. The HBP group at 300 µg/mL showed efficacy closest to that of the positive control group.

### 3.5. Immunofluorescence Analysis of C2C12 Myogenic Differentiation and Myotube Formation

**Figure 4:**
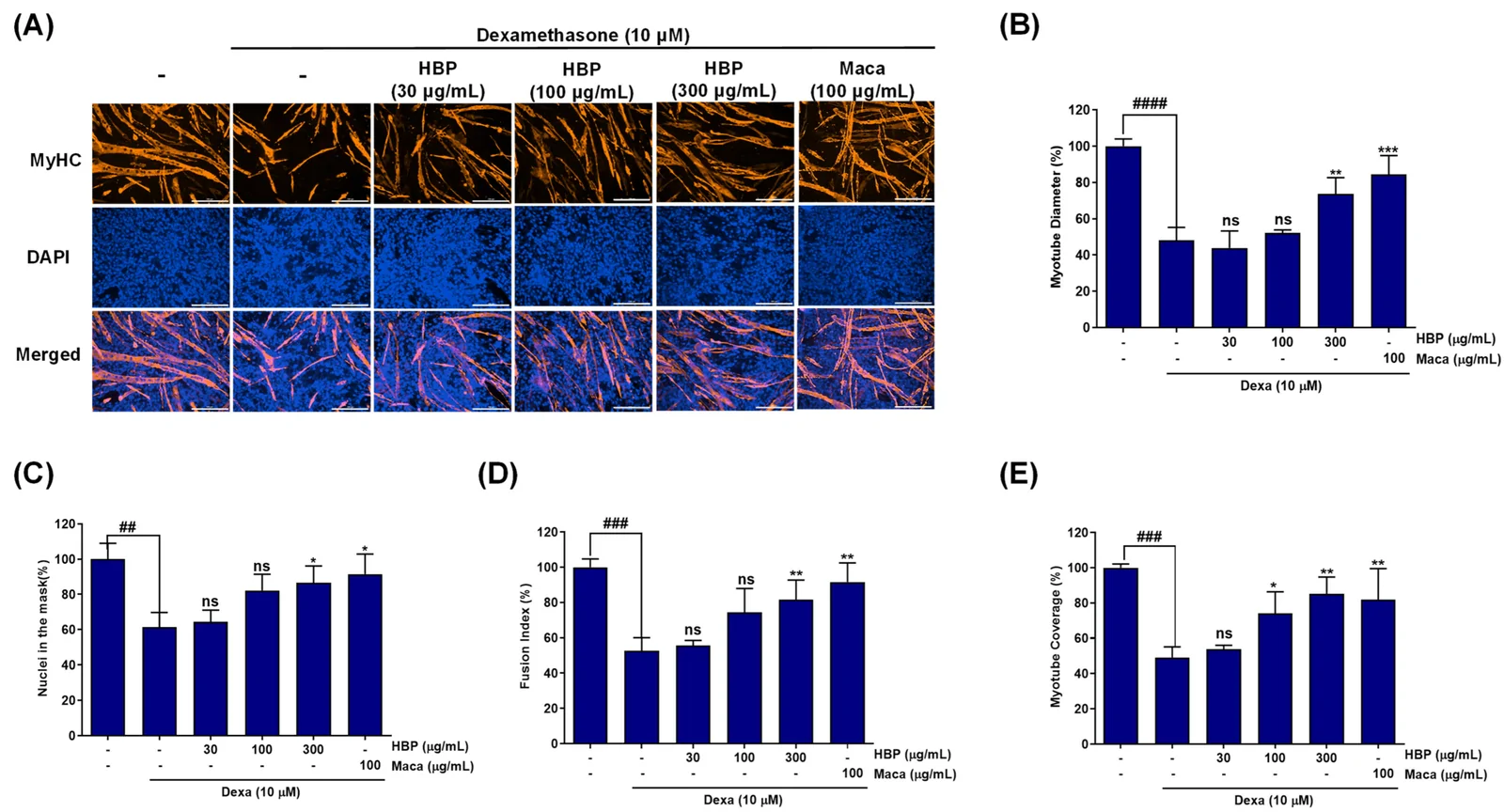
Effect of HBP on MyHC expression and myotube formation during dexamethasone (Dexa) exposure initiated at the onset of C2C12 differentiation. (**A**) MyHC immunofluorescence images; scale bars = 200 µm. (**B**) Myotube diameter. (**C**) Nuclei within the MyHC-positive mask. (**D**) Fusion index. (**E**) Myotube coverage. Data are expressed as the mean ± SD of three independently acquired replicate images per treatment group (*n* = 3). Each replicate image was analyzed separately using Myotube Analyzer, and one image-level value for each endpoint was used in the statistical analysis. Statistical significance was evaluated using one-way ANOVA followed by Dunnett’s multiple-comparisons test, with the Dexa-treated group used as the reference group. Significant differences were identified at * *p* < 0.05, ** *p* < 0.01 and *** *p* < 0.001 as compared to the HBP control; ## *p* < 0.01, ### *p* < 0.001 and #### *p* < 0.0001 as compared to the DEXA-induced control; ns means are not significant.

MyHC immunofluorescence staining was performed to evaluate myogenic differentiation in HBP-treated C2C12 cells. MyHC immunofluorescence images and quantitative measures of myotube diameter, nuclei within the MyHC-positive mask, fusion index, and myotube coverage are presented in Figure 4A. Compared with the control group, dexamethasone treatment resulted in thinner and less uniformly formed myotubes. HBP progressively affects myotube morphology in a concentration-dependent manner, with thicker and more uniformly formed myotubes observed at higher HBP concentrations. Quantitative analyses support these morphological observations (Figure 4B–E). The number of nuclei within the MyHC-positive mask (Figure 4C) and fusion index (Figure 4D) were reduced by approximately 50% following dexamethasone treatment. Both parameters increased with increasing HBP concentration, with significant increases observed at 300 µg/mL. Myotube diameter (Figure 4B) and myotube coverage (Figure 4E) were also significantly increased by HBP treatment compared with dexamethasone treatment, with values approaching those of the control group, although no direct statistical comparison with the control group was performed for this specific endpoint. At 300 µg/mL, HBP produced responses approaching those observed in the positive control, Maca (100 µg/mL). Collectively, these findings indicate that HBP attenuated dexamethasone-induced impairment of C2C12 myogenic differentiation and myotube formation. Because HBP and dexamethasone treatment began at the onset of differentiation, these findings should be interpreted as attenuation of dexamethasone-associated impairment during myogenic differentiation and myotube formation, rather than reversal of established atrophy in mature myotubes.

## 4. Discussion

Previous studies of hairtail-derived protein hydrolysates have mainly focused on the isolation and characterization of antioxidant peptides [2], whereas their potential effects on muscle atrophy remain poorly understood. In the present study, protein hydrolysates prepared from hairtail byproducts improved cell viability, myotubular formation, and myogenic differentiation in a dexamethasone-induced C2C12 muscle atrophy model. These findings provide preliminary evidence supporting the potential use of hairtail byproducts as a source of functional protein hydrolysates for muscle health. Importantly, HBP and dexamethasone were applied from the onset of differentiation. Therefore, the present design does not demonstrate reversal of established atrophy in mature myotubes and should instead be interpreted as testing preservation of myogenic differentiation and myotube formation during glucocorticoid exposure.

The crude protein content of the hairtail byproducts was 53.58%, consistent with the relatively high protein content reported for other fish species. Previous studies have reported crude protein contents of approximately 40–51% in fish byproducts, including *Lethrinus lentjan*, *Stolephorus indicus*, *Sardinella longiceps*, and *Lates calcarifer* [42]. These findings support the suitability of hairtail byproducts as a protein-rich raw material for enzymatic hydrolysis and the production of functional peptides.

The protein content of the resulting HBP was 82.4%, which falls within the approximately 60–90% range reported for fish protein hydrolysates in previous studies [39,43]. Together with the higher extraction yield obtained by Protamex-assisted hydrolysis compared with water extraction, this finding suggests that enzymatic hydrolysis was effective for recovering protein-rich material from hairtail byproducts.

Aromatic amino acids, including tryptophan, phenylalanine, and tyrosine, can contribute to antioxidant activity through the electron- or hydrogen-donating properties of their aromatic side chains [44,45]. The amino acid profile of HBP showed relatively low levels, with tyrosine at 1.84 g/100 g and phenylalanine at 3.09 g/100 g, and tryptophan was not detected. These figures raise the question of whether HBP’s antioxidant activity can be explained by aromatic amino acids alone. Therefore, it should be noted that the antioxidant activity of protein hydrolysates is not determined solely by the total content of aromatic amino acids, but is influenced by various factors such as the type, arrangement order, and position of amino acid residues within individual peptides, together with the overall molecular weight distribution of the hydrolysates [41]. Excessive ROS production activates proteolytic pathways involved in skeletal muscle atrophy, including FoxO-mediated induction of MuRF-1 and Atrogin-1. Accordingly, aromatic amino acid content of HBP may contribute to the reduction in oxidative stress and thereby potentially attenuate pathways associated with muscle protein degradation. This study has the limitation of not being able to conduct experiments to confirm this mechanism, so further research is needed to determine whether this pathway is involved.

HBP exhibited concentration-dependent ABTS and DPPH radical-scavenging activities, indicating antioxidant potential. However, the ABTS IC_50_ value of the HBP obtained in this study was 5.653 mg/mL, and the DPPH radical scavenging IC_50_ value was 1.933 mg/mL, which is relatively higher than that of hydrolysis products or purified peptides derived from other fish. According to other research results, the ABTS IC_50_ values of hydrolysates of cod, herring, and mackerel were reported to vary between approximately 2.76 and 125.25 mg/mL, and the DPPH IC_50_ values ranged between approximately 1.74 and 22.82 mg/mL [46].

In another study, fractions of hydrolyzed tuna byproduct larger than 4 kDa showed only about 10% of low DPPH antioxidant activity, while smaller fractions exhibited higher antioxidant activity of about 50~60%. Through this, unlike the case using hydrolysis, additional separation and purification were carried out [47]. Therefore, the HBP used in this experiment is estimated to have a higher IC_50_ value than that obtained by the low-molecular-weight fractionation method. Previous studies similarly showed that the antioxidant properties of fish protein hydrolysates depend on the presence and position of specific amino acids within peptide sequences [48,49]. In addition, peptides with a small molecular weight of 1~5 kDa are generally reported to contain stronger antioxidant peptides [50]. Thus, the antioxidant activity observed for HBP remains to be established in future work.

HBP was high in aspartic acid (15.82%) and glutamic acid (15.66%). Aspartic acid, through the tricarboxylic acid cycle, also supports glutathione synthesis via α-ketoglutarate production, and through its conversion to β-alanine, a precursor of carnosine, which directly scavenges hydroxyl radicals and peroxynitrite [31]. Glutamine has been reported as a precursor to glutathione metabolism and as having antioxidant effects [51]. In particular, the effect of suppressing glutamate-dependent myostatin to protect muscles in mouse glucocorticoid-induced atrophy [52] and that taking glutamic acid as a dietary supplement together with arginine appears to reduce the expression of muscle atrophy-related genes MuRF1 and Atrogin-1 in skeletal muscle [53]. Taken together with its abundant glutamic acid content, these findings suggest that the amino acid composition of HBP may collectively contribute to both its antioxidant and anti-atrophic potential. The antioxidant efficacy of protein hydrolysates is influenced by peptide size, amino acid composition, sequence, and the accessibility of hydrophobic and aromatic residues. However, this study has the limitation of not conducting peptide sequence analysis. Therefore, if further research is conducted, it is considered necessary to carry out degree of hydrolysis, MW distribution, and peptide sequence analysis, including addition. The antioxidant and antiatrophic activities reported here should be interpreted as properties of the HBP mixture as tested, rather than being attributed to specific peptides.

In the dexamethasone-induced C2C12 model, HBP did not exhibit cytotoxicity at the tested concentrations and was associated with concentration-dependent increases in cell viability. These findings indicate that HBP was well tolerated under the experimental conditions and support the interpretation of the subsequent differentiation findings without evidence of cytotoxicity. HBP also increased cell proliferation in a concentration-dependent manner [54]. In general, skeletal muscle growth and protein synthesis are regulated in part through the PI3K/Akt/mTOR pathway, which promotes downstream signaling through targets such as S6K1 and 4E-BP1 [55,56]. However, although ABTS and DPPH assays confirm the chemical radical-scavenging capacity of protein hydrolysates in this study, they do not directly demonstrate the reduction in oxidative stress or attenuation of muscle atrophy within living cells. Recent studies have shown that intracellular ROS and protein carbonylation increase alongside muscle atrophy in C2C12 myotubes, mediated in part by downregulation of the Nrf2/HO-1 pathway [57]. A limitation of the present study is that its antioxidant evaluation relied solely on ABTS and DPPH chemical radical-scavenging assays. This study did not measure intracellular ROS levels, antioxidant enzyme activity, oxidative damage markers, Nrf2/HO-1 signaling, or the atrophy-related markers MuRF1, Atrogin-1/MAFbx, and Akt/FoxO/mTOR signaling. Cell-based validation, such as measurement of intracellular ROS, oxidative damage markers, and redox-related signaling pathways, was not performed. Future work should therefore assess these cellular endpoints directly in a muscle cell model to establish a mechanistic link between the antioxidant activity of HBP and its protective effects against muscle atrophy.

MyHC immunofluorescence analysis showed concentration-dependent improvements in myotube morphology and quantitative indices of myogenic differentiation following HBP treatment. Dexamethasone reduced myotube diameter, nuclei within the MyHC-positive area, fusion index, and myotube coverage, whereas these parameters increased with HBP treatment, particularly at 300 µg/mL. These findings indicate that HBP attenuated dexamethasone-induced impairment of myotube formation and myogenic differentiation. The mitogen-activated protein kinase pathway has been implicated in skeletal muscle differentiation [58,59], although the involvement of this pathway in the effects of HBP was not examined directly. This interpretation is constrained by the treatment schedule: because HBP and dexamethasone were introduced when differentiation began, the observed morphology reflects protection against dexamethasone-associated impairment during myotube development rather than recovery of already established myotube atrophy.

Oxidative stress and chronic inflammation are important contributors to skeletal muscle atrophy [60]; HBP contained high levels of leucine and arginine, as well as aromatic amino acids that may contribute to antioxidant activity. Previous studies reported muscle-protective effects of naturally derived protein hydrolysates. For example, Spirulina platensis protein hydrolysates were reported to promote muscle differentiation and protein synthesis in dexamethasone-induced atrophy models, whereas hydrolysates derived from flounder (*Paralichthys olivaceus*), mackerel, and oysters demonstrated antioxidant and muscle-protective effects involving muscle atrophy-related pathways [61,62,63,64]. The present findings extend this literature by suggesting that hairtail byproducts represent another marine source of protein hydrolysates with potential muscle-protective activity.

HBP contained 6.80% arginine and 5.81% leucine, both of which are used in pathways involved in muscle synthesis [65]. Phenylalanine and tyrosine accounted for 4.12% and 2.45% of the constituent amino acids, respectively, whereas the free amino acids L-phenylalanine, L-tyrosine, and L-tryptophan accounted for 4.77%, 3.25%, and 2.79%, respectively. Leucine is a branched-chain amino acid that promotes muscle protein synthesis through mTORC1 signaling, whereas arginine has been implicated in mTOR signaling and nitric oxide production. These amino acids may therefore have contributed to the biological effects observed with HBP; however, their individual contributions cannot be determined from the present experiments.

Recent studies have further expanded the landscape of marine-derived anti-atrophic hydrolysates, though with notable differences in experimental scope. Kurera et al. (2024) demonstrated that oyster (*Crassostrea nippona*) hydrolysates attenuated dexamethasone-induced atrophy in C2C12 cells via modulation of MuRF-1, Atrogin-1, FoxO3a, MyoD, and mTOR, but their evaluation was limited to gene/protein expression without an in vivo model [66]. Park and Lee (2025) reported that mackerel protein hydrolysates protected C2C12 myotubes against oxidative stress-induced atrophy through Akt/FoxO activation and NF-κB suppression, but this study employed an oxidative stress rather than a glucocorticoid-induced atrophy model and likewise lacked in vivo validation [64]. In contrast, Hyun et al. (2025) and Lee et al. (2026) provided more comprehensive evaluations of olive flounder (*Paralichthys olivaceus*) byproduct hydrolysates, combining C2C12 assays (myotube diameter, fusion index) with in vivo validation in zebrafish and rodent models, respectively, along with lipidomic profiling of membrane phospholipid composition, an endpoint not addressed in any of the aforementioned studies, including the present one [62,67]. Compared with these species, the present study is, to our knowledge, the first to characterize the anti-atrophic potential of a byproduct-derived hydrolysate from hairtail (*Trichiurus* spp.). Collectively, these studies illustrate that while cell-based evidence for the anti-atrophic potential of marine hydrolysates is accumulating rapidly, in vivo validation and mechanistic depth remain inconsistent across species, underscoring both the relevance of the present in vitro findings on hairtail-derived HBP and the need for future in vivo confirmation to reach the standard now being set by olive flounder-derived studies.

Overall, HBP prepared from hairtail byproducts exhibited a high protein content, contained several amino acids with potential biological relevance, and demonstrated antioxidant activity. In addition, in dexamethasone-treated C2C12 cells, HBP was associated with increased cell viability, improved myotube formation, and enhanced indices of myogenic differentiation. These results suggest that HBP may have potential as a functional protein material for further investigation in muscle health. In addition, valorization of hairtail processing byproducts may provide an opportunity to increase the value of marine processing residues and support more sustainable utilization of fishery resources. Further studies are required to identify the active peptides, clarify the molecular mechanisms involved, and determine whether these effects can be reproduced in animal and human studies. Accordingly, the cell-based findings should be regarded as preliminary evidence from a concurrent differentiation/dexamethasone-exposure model rather than evidence that HBP restores pre-existing muscle atrophy.

## 5. Conclusions

HBP prepared from hairtail byproducts exhibited a high protein content, contained several amino acids of potential biological relevance, and demonstrated ABTS and DPPH radical-scavenging activity. In C2C12 cells exposed to dexamethasone from the onset of differentiation, HBP was associated with increased cell viability and proliferation, as well as improved indices of myogenic differentiation and myotube formation. Because HBP and dexamethasone were applied throughout differentiation, these findings should be interpreted as attenuation of dexamethasone-associated impairment during myogenic differentiation rather than reversal of established atrophy in mature myotubes. Protamex-assisted hydrolysis yielded approximately three times more recovered material than water extraction under the tested conditions; no economic or scale-up analysis was performed. Valorization of hairtail processing byproducts may provide an opportunity to increase the value of marine processing residues and support more sustainable utilization of fishery resources. Collectively, the present findings indicate that HBP warrants further investigation as a candidate functional protein ingredient for supporting muscle health. Further studies should characterize peptide composition, clarify the underlying molecular mechanisms, and determine whether these effects are reproducible in animal and human studies.

## Figures and Tables

**Table 1:** Proximate (%) of hairtail (*T. lepturus*) by-products.

	Proximate Composition (%)			
Hairtail Byproduct	Moisture	Crude Protein	Crude fat	Crude Ash
	7.15 ± 0.16	53.58 ± 1.80	19.96 ± 0.77	17.74 ± 0.21

**Table 2:** Constituent amino acid composition of HBP.

Constituent Amino Acid	Composition (g/100 g)	Percentage (%)
Aspartic acid	11.84	15.82
Threonine	4.33	5.79
Serine	4.08	5.46
Glutamic acid	11.72	15.66
Proline	4.46	5.96
Glycine	4.96	6.63
Alanine	4.34	5.80
Cystine	0.41	0.54
Valine	2.69	3.60
Methionine	2.73	3.65
Isoleucine	3.41	4.56
Leucine	4.35	5.81
Tyrosine	1.84	2.45
Phenylalanine	3.09	4.12
Histidine	1.71	2.29
Lysine	3.82	5.10
Arginine	5.06	6.80
Total	74.84	100.00

**Table 3:** Free amino acid composition of HBP.

Free Amino Acid	Composition (g/100 g)	Percentage (%)
O-Phospho-L-serine	N.D *	N.D
Taurine	5.03	6.36
O-Phosphoethanolamine	N.D	N.D
Urea	2.32	2.93
L-Aspartic Acid	1.05	1.33
Hydroxy-L-Proline	0.23	0.29
L-Threonine	2.79	3.52
L-Serine	1.73	2.19
L-Asparagine	2.44	3.08
L-Glutamic Acid	3.30	4.17
L-Sarcosine	0.70	0.88
L-α-Aminoadipic Acid	0.27	0.34
L-Proline	2.65	3.35
Glycine	0.94	1.18
L-Alanine	3.80	4.79
L-Citrulline	0.79	1.00
L-α-Aminoiso-n-butyric Acid	0.16	0.21
L-Valine	2.94	3.71
L-Cystine	0.12	0.15
L-Methionine	2.95	3.72
Cystathionine-1	3.47	4.38
Cystathionine-2	2.28	2.88
L-Isoleucine	2.21	2.80
L-Leucine	9.48	11.97
L-Tyrosine	2.57	3.25
β-Alanine	2.41	3.05
L-Phenylalanine	3.78	4.77
D,L-β-Aminoisobutyric Acid	0.17	0.22
L-Homocystine	1.26	1.59
γ-Amino-n-butyric Acid	0.88	1.11
Ethanolamine	1.30	1.64
δ-Hydroxylysine	2.13	2.69
L-Ornithine	1.01	1.27
L-Lysine	2.06	2.60
1-Methyl-L-histidine	0.95	1.20
L-Histidine	0.88	1.11
L-Tryptophan	2.21	2.79
3-Methyl-L-histidine	N.D	N.D
L-Anserine	0.49	0.62
L-Carnosine	2.64	3.34
L-Arginine	2.80	3.54
Total	79.18	100.00

* Not detected.

## Data Availability

The original contributions presented in this study are included in the article. Further inquiries can be directed to the corresponding authors.

## Abbreviations

The following abbreviations are used in this manuscript:

HBP	Hairtail Byproducts Protamex-assisted enzymatic hydrolysate
MyHC	Myosin Heavy Chain
DAPI	4′,6-diamidino-2-phenylindole
ROS	Reactive Oxygen Species
ABTS	2,2′-Azino-Bis(3-ethylbenzothiazoline-6-sulfonic acid)
DPPH	2,2-Diphenyl-1-picrylhydrazyl
Dexa	Dexamethasone
MTT	3-(4,5-Dimethylthiazol-2-yl)-2,5-Diphenyltetrazolium Bromide
BrdU	Bromodeoxyuridine
mTOR	Mechanistic Target of Rapamycin
ECM	Extracellular Matrix
